# Site-Specific Expression of Gelatinolytic Activity during Morphogenesis of the Secondary Palate in the Mouse Embryo

**DOI:** 10.1371/journal.pone.0047762

**Published:** 2012-10-16

**Authors:** Nikolaos Gkantidis, Susan Blumer, Christos Katsaros, Daniel Graf, Matthias Chiquet

**Affiliations:** 1 Department of Orthodontics and Dentofacial Orthopedics, School of Dental Medicine, University of Bern, Bern, Switzerland; 2 Institute for Oral Biology, Center for Dental Medicine, University of Zurich, Zurich, Switzerland; Ecole Normale Supérieure de Lyon, France

## Abstract

Morphogenesis of the secondary palate in mammalian embryos involves two major events: first, reorientation of the two vertically oriented palatal shelves into a horizontal position above the tongue, and second, fusion of the two shelves at the midline. Genetic evidence in humans and mice indicates the involvement of matrix metalloproteinases (MMPs). As MMP expression patterns might differ from sites of activity, we used a recently developed highly sensitive in situ zymography technique to map gelatinolytic MMP activity in the developing mouse palate. At embryonic day 14.5 (E14.5), we detected strong gelatinolytic activity around the lateral epithelial folds of the nasopharyngeal cavity, which is generated as a consequence of palatal shelf elevation. Activity was concentrated in the basement membrane of the epithelial fold but extended into the adjacent mesenchyme, and increased in intensity with lateral outgrowth of the cavity at E15.5. Gelatinolytic activity at this site was not the consequence of epithelial fold formation, as it was also observed in *Bmp7*-deficient embryos where shelf elevation is delayed. In this case, gelatinolytic activity appeared in vertical shelves at the exact position where the epithelial fold will form during elevation. *Mmp2* and *Mmp14* (MT1-MMP), but not *Mmp9* and *Mmp13*, mRNAs were expressed in the mesenchyme around the epithelial folds of the elevated palatal shelves; this was confirmed by immunostaining for MMP-2 and MT1-MMP. Weak gelatinolytic activity was also found at the midline of E14.5 palatal shelves, which increased during fusion at E15.5. Whereas MMPs have been implicated in palatal fusion before, this is the first report showing that gelatinases might contribute to tissue remodeling during early stages of palatal shelf elevation and formation of the nasopharynx.

## Introduction

Cleft lip with or without cleft palate (CLP) and cleft palate only (CPO) are among the most frequent birth defects in humans, affecting 1–2 per thousand newborns [1], [2], [3], [4]. About 70% of cases are considered non-syndromic, whereas the remainder is associated with various other defects [5]. A multitude of genetic as well as environmental factors are believed to contribute to the etiology of CLP and CPO [2]. However, in most of the non-syndromic cases the cause remains unknown [5]. In mouse knockout models, close to 20 genes have been identified that upon deletion result in cleft palate; many of them code for components of the TGF-β, BMP, WNT, and FGF signaling pathways [6], [7]. Morphogenesis of the secondary palate is a complex process involving several consecutive events, which occur from embryonic day E13.5 to E15.5 in the mouse and between the 6th and 10th week of gestation in humans [8], [9]. First, paired palatal shelves grow out vertically from the maxillary processes on both sides of the tongue. In a second step, the palatal shelves elevate into a horizontal position above the tongue. As a consequence, two opposing epithelial folds (one from each shelf) are generated comprising the lateral margins of a newly formed cavity, which will eventually give rise to the nasopharynx. The palatal shelves grow horizontally and contact each other, forming the midline epithelial seam (MES). Finally, the medial edge epithelial (MEE) cells disappear, and the two palatal shelves fuse by establishing continuity between their mesenchyme [8]. Through this morphogenetic process, the secondary palate separates the primary oral cavity into the oropharynx and the nasopharynx, supporting vital functions such as feeding and breathing. Cleft palate can arise from either a deficit of the palatal shelves to grow to appropriate size, from disturbances in palatal shelf elevation, or from a lack of shelf fusion [7].

Experiments in mice [10], [11], [12], [13] and genetic linkage studies in humans [14], [15] indicate that, besides defects in growth factor signaling, deficiencies in certain matrix metalloproteinases (MMPs) are involved in the etiology of cleft lip and palate. MMPs comprise a family of secreted Zn^2+^-dependent proteases that cleave extracellular matrix (ECM) proteins and are known to be essential for tissue remodeling in development and disease [16]. Specific domains of MMPs provide substrate specificity [17], [18]: for example, the “collagenases” MMP-1, −8, and −13 mainly attack native fibrillar collagens, whereas MMP-2 and MMP-9 are the major “gelatinases” that cleave partially denatured collagen, as well as collagen IV in basement membranes [18], [19]. Most MMPs are secreted as inactive proenzymes and their activity is regulated in intricate ways, positively by proteolytic cleavage of the propeptide [20] and negatively (in most cases) by tissue inhibitors of metalloproteinases (TIMPs; [21]). Membrane-type MMPs (e.g. MT1-MMP/MMP-14) on cell surfaces are required for activation of major secreted MMPs (e.g. MMP-2/gelatinase B) [20].

**Figure 1:**
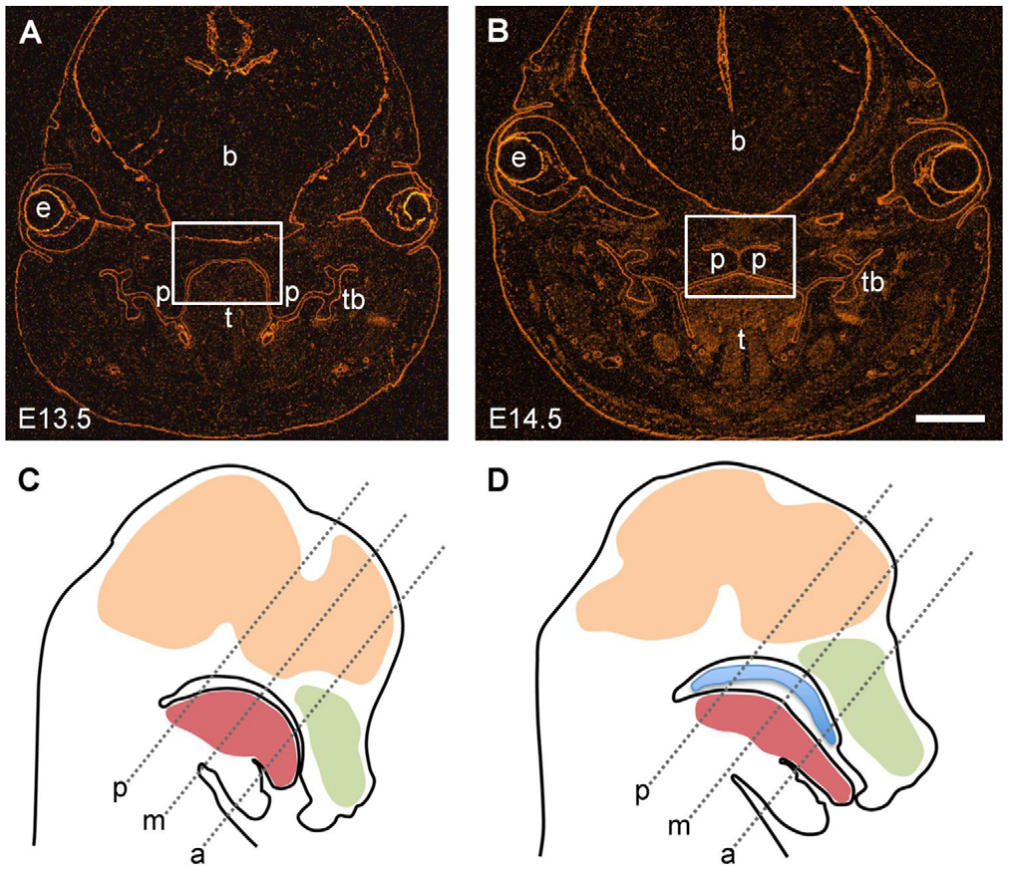
Overview of craniofacial structures in the mouse embryo. Frontal sections (mid-posterior level) of (A) E13.5 and (B) E14.5 wild type mouse heads were labeled with antibody to laminin-111, which stains all basement membranes. The boxed areas illustrate the region of interest that is presented in most of the following figures. Note that at E13.5 the palatal shelves are vertically oriented besides the tongue, while at E14.5 the shelves have elevated above the tongue to form the secondary palate. e, eye; b, forebrain; t, tongue; p, palatal shelf; tb, tooth buds. Bar, 500 μm. Drawings of E13.5 (C) and E14.5 (D) mouse heads indicate the different anteroposterior section planes through the palatal region (a, anterior; m, middle; p, posterior) that are presented in following figures. Note that at E14.5, the elevated secondary palate now separates the oral from the newly formed nasopharyngeal cavity.

MMPs have been implicated in the process of palatal shelf fusion. Palatal shelves from E14.0 wild type mouse embryos failed to fuse when cultured in close proximity in the presence of synthetic MMP inhibitor or excess TIMP-2 [12]. In this case, MMPs are likely to function downstream of TGF-β3, a central regulator of palatal shelf fusion in mice and humans [5], [14], [22], [23], [24]. *Tgfβ3*-deficient mouse embryos expressed lower levels of MMP-2 (gelatinase B) and MMP-13 (collagenase 3) in the developing palate, and addition of TGF-β3 to palatal mesenchyme cultures induced MMP-13 expression [12]. Similarly, MT6-MMP (MMP-25), which has been linked to cleft palate in humans, is also regulated by TGF-β3 and required for palatal shelf fusion in vitro [25]. These results demonstrate that tissue remodeling by specific MMPs plays an important role in the late steps of secondary palate formation.

**Figure 2:**
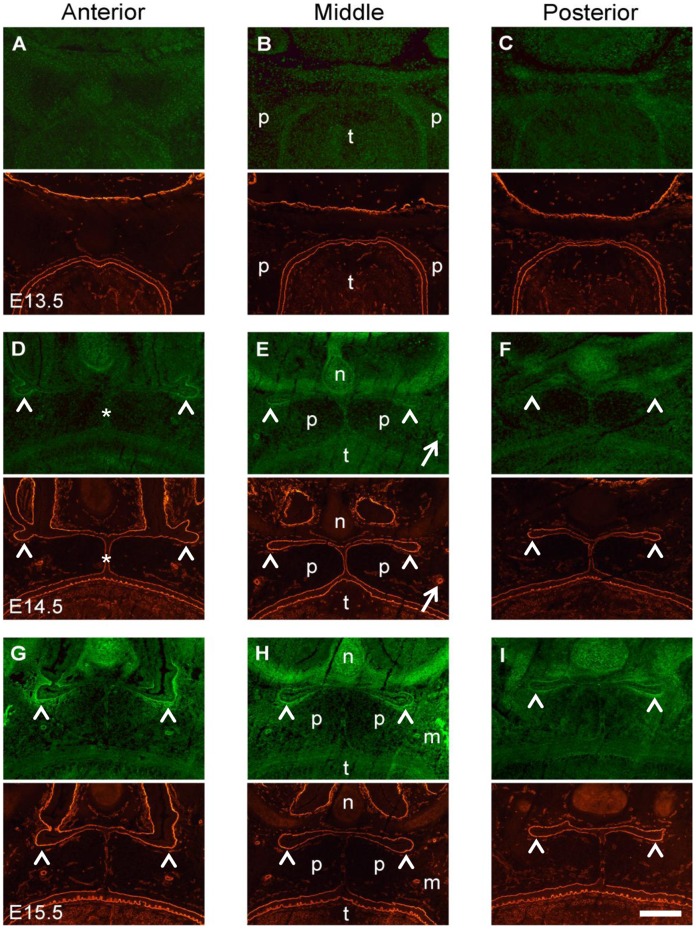
Double labeling for gelatinolytic activity and laminin in the palatal region of the mouse embryo. Frontal cryosections of E13.5 (A–C), E14.5 (D–F), and E15.5 (G–I) wild type mouse heads were subjected to DQ-gelatin zymography (green panels), followed by immunofluorescence labeling for laminin-111 on the same section (red panels). The images show representative sections from the anterior (A, D, G), the middle (B, E, H), and the posterior (C, F, I) level of the palate. No site with significant gelatinolytic activity, as manifested by increased fluorescence, is detected at E13.5 prior to palatal shelf elevation. At E14.5, gelatinolytic activity is evident in the nasal cartilage and in the main palatal arteries (arrows). In addition, signs of gelatinolysis are visible at the midline epithelial seam (asterisks), whereas prominent gelatinolytic activity is detected around the folds of the elevated palatal shelves, where the nasopharynx will form (arrowheads). A similar pattern of gelatinolysis is observed at E15.5, although activity is increased compared to E14.5. Furthermore, prominent activity was evident for maxillary bone. p, palatal shelf; t, tongue; n, nasal cartilage; m, maxillary bone. Bar, 200 μm.

Compared to palatal shelf fusion, little is known yet about the molecular mechanism of palatal shelf elevation. It has been proposed that changes in osmotic pressure due to adsorption of water by accumulating hyaluronic acid causes shelf elevation [8], [26], [27], although direct evidence is missing. A mechanism by which the vertical shelves simply swing up by 90° around an imaginary “hinge” seems unlikely, as substantial reorganization of mesenchymal tissue is documented during shelf elevation. The presumptive middle edge epithelial (MEE) cells are not located exactly at the apex of the vertical shelves, but instead on their distal-medial aspect facing the tongue [28]. Moreover, tracking experiments indicated that during shelf elevation, the lower half of the vertical shelves bulges out mesially, whereas part of the upper half retracts laterally, forming an epithelial fold [29]. Thus, like for other morphogenetic events, coordinated cell contractions/movements and remodeling of the extracellular matrix are likely mechanisms underlying palatal shelf elevation [30]. Here, we used a highly sensitive in situ zymography technique to detect gelatinolytic activity in palatal shelves of mouse embryos at the stage of their elevation and fusion. The method is based on incubating unfixed cryosections with dye-quenched gelatin (DQ-gelatin) whose fluorescence increases upon enzymatic cleavage [31]. The major enzymes detected in tissues by gelatin zymography are MMP-2 (gelatinase B) and MMP-9 (gelatinase A); however other MMPs with weaker gelatinolytic activity might contribute to the signal [32]. This method is able to detect gelatinolytic activity associated with minute structures such as distinct basement membranes in the developing mouse embryo head [33]. Double labeling with antibodies to specific MMPs allows to reveal the relationship between active enzyme and protein distribution, which might differ due to the complex regulation of MMP activity.

**Figure 3:**
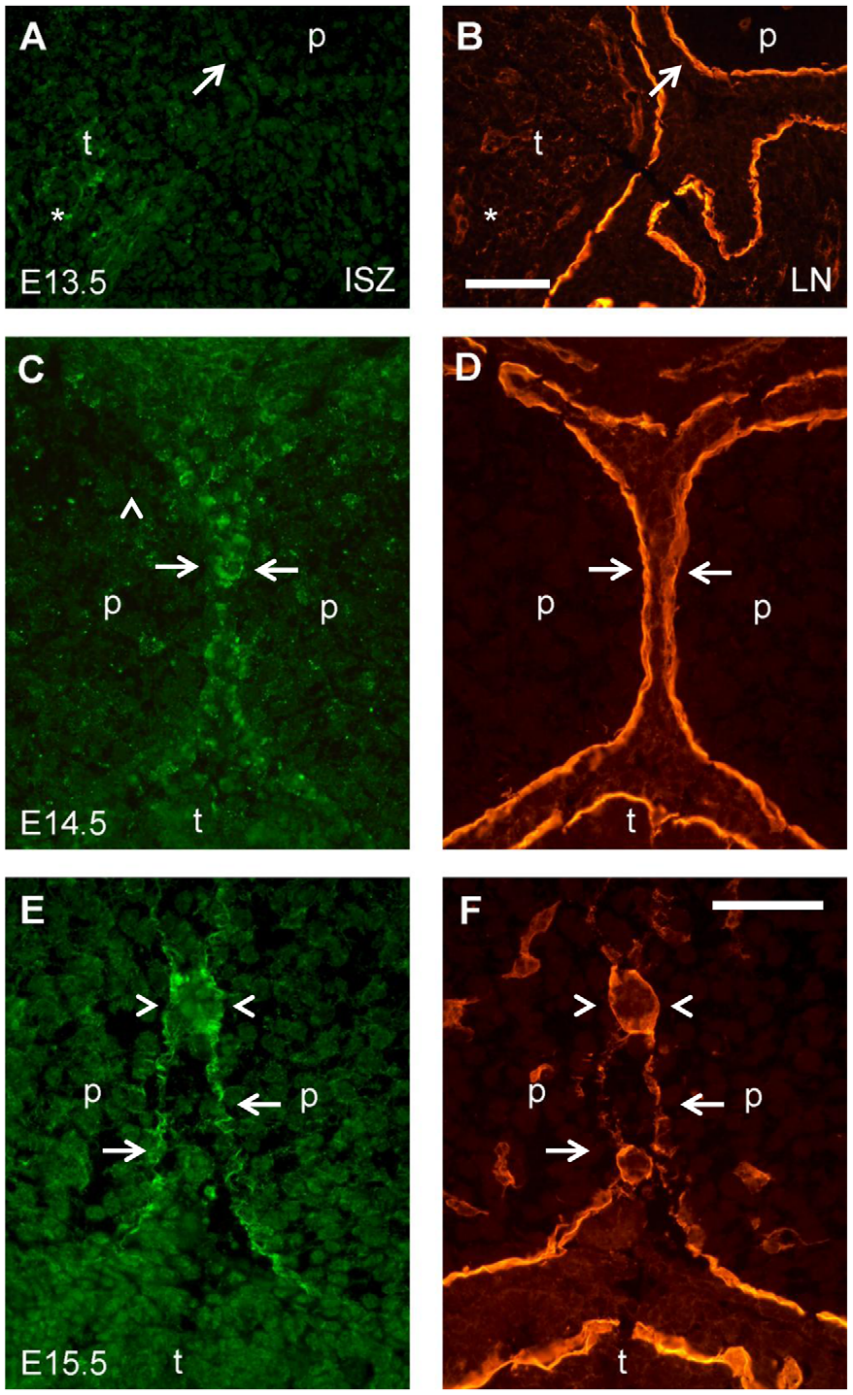
Close-up images of double labeling for gelatinolytic activity and laminin around medial edge epithelial cells of palatal shelves. Frontal cryosections (middle anteroposterior level) from E13.5 (A, B), E14.5 (C, D), and E15.5 (E, F) wild type mouse heads were subjected to DQ-gelatin zymography, followed by immunofluorescence labeling for laminin on the same section. (A) In situ zymography and (B) laminin labeling, respectively, in the distal-medial region of a E13.5 palatal shelf, where epithelial fusion will occur after shelf elevation. No sign of activity was detected at that site (arrows), while gelatinolysis was evident in the tongue mesenchyme (asterisks). (C) In situ zymography and (D) laminin labeling, respectively, immediately prior to midline fusion of the palatal shelves at E14.5. Note weak gelatinolytic activity at the site where epithelial fusion will occur (arrows). (E) In situ zymography and (F) laminin labeling, respectively, during midline fusion of the palate at E15.5. Prominent gelatinolytic activity is evident at sites of midline epithelial fusion (arrows), and at the epithelial remnants of the palatal shelves at the midline (arrowheads). p, palatal shelf; t, tongue. Bars, 50 μm.

We provide the first evidence that gelatinases are involved not only in the process of palatal shelf fusion but also in their elevation, as gelatinolytic activity was detected in the basement membrane and underlying mesenchyme of the nasopharyngeal folds that formed when shelves reoriented from vertical to horizontal position. In a model of delayed palatal shelf elevation, the still vertically oriented shelves displayed gelatinolytic activity at the exact site where the epithelial fold would normally form. This suggested that the marked increase in gelatinolytic activity at this position might be causally involved in the formation of the epithelial fold during shelf elevation, rather than being a consequence of the process.

**Figure 4:**
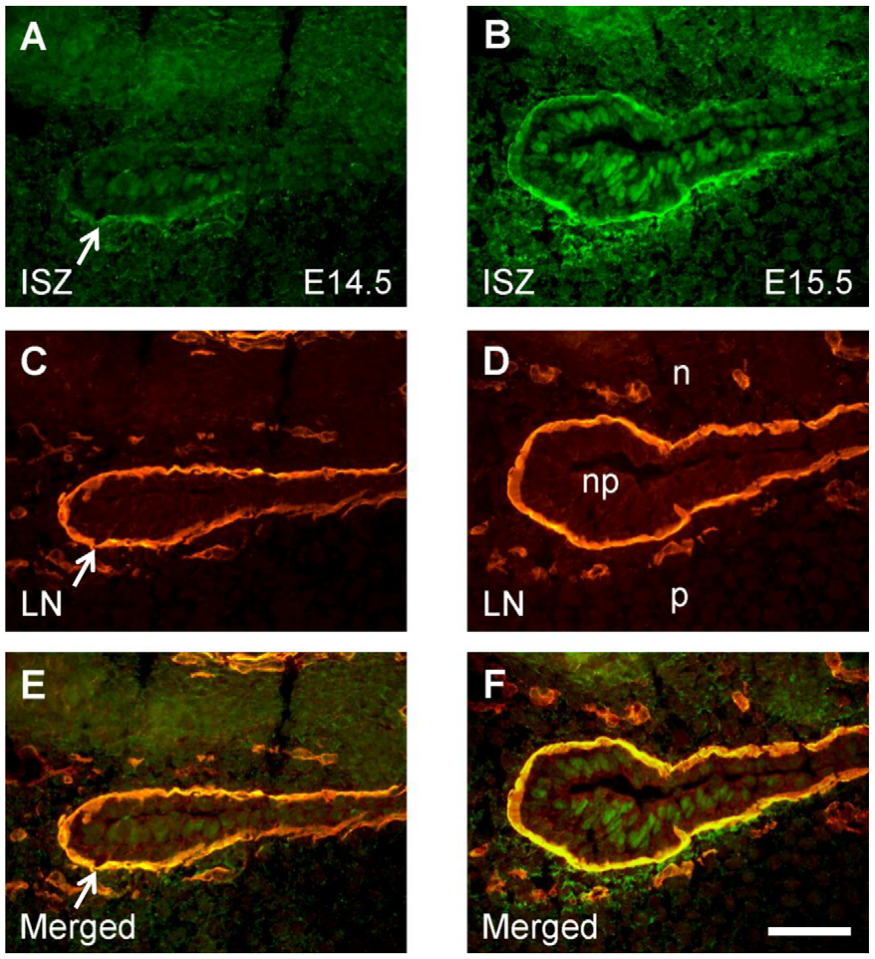
Enlarged images of the palatal-nasopharyngeal fold after double labeling for gelatinolytic activity and laminin. Frontal cryosections at the middle anteroposterior level of E14.5 (A, C, E) and E15.5 (B, D, F) wild type mouse heads were subjected to DQ-gelatin in situ zymography (ISZ; A, B), followed by immunofluorescence labeling for laminin-111 (LN; C, D) on the same section. Merged images are shown in E and F. At E14.5, gelatinolytic activity colocalizes with the epithelial basement membrane at the lateral and palatal side of the fold (arrows). The pattern of gelatinolysis is similar at E15.5, although activity extends into the adjacent mesenchyme and its intensity is considerably increased. p, palatal shelf; n, nasal floor; np, nasopharynx. Bar, 50 μm.

## Materials and Methods

### Animals, embryonic tissue, and cryosectioning

Animal experiments were approved by the local veterinary authorities (permit 98/2011, Veterinäramt Zürich) in compliance with Swiss federal law (TSchG, TSchV) and cantonal by-laws in full compliance with the European Guideline 86/609/EC. This authority approval also included ethical approval. C57BL/6 wildtype mouse embryos were obtained from J.-F. Spetz at the Friedrich-Miescher Institute for Biomedical Research (FMI) in Basel, Switzerland. Embryos were obtained by timed mating, and E0.5 was considered as the morning where the vaginal plug was seen. Pregnant females were sacrificed by cervical dislocation at the desired stage (E13.5, E14.5, and E15.5), embryos were harvested and decapitated. The *Bmp7* null allele of the *Bmp7* heterozygous null mice was generated by Cre-mediated recombination in the germ line of a conditional *Bmp7* allele (*Bmp7^flx^*), in which exon 1 is flanked by loxP sites as described earlier [34]. *Bmp7* heterozygous null mice (*Bmp7^+/Δ^*) were intercrossed to obtain *Bmp7^Δ/Δ^* and control embryos from the same litter. E14.5 embryos were harvested from timed pregnant mice sacrificed by cervical dislocation. Genotyping of embryos was carried out by allele-specific PCR [34]. In total, 6 E13.5, 14 E14.5, and 12 E15.5 C57BL/6 wild type, as well as 4 *Bmp7^+/Δ^ and 4 Bmp7^Δ/Δ^* mouse embryos were used for the present study.

**Figure 5:**
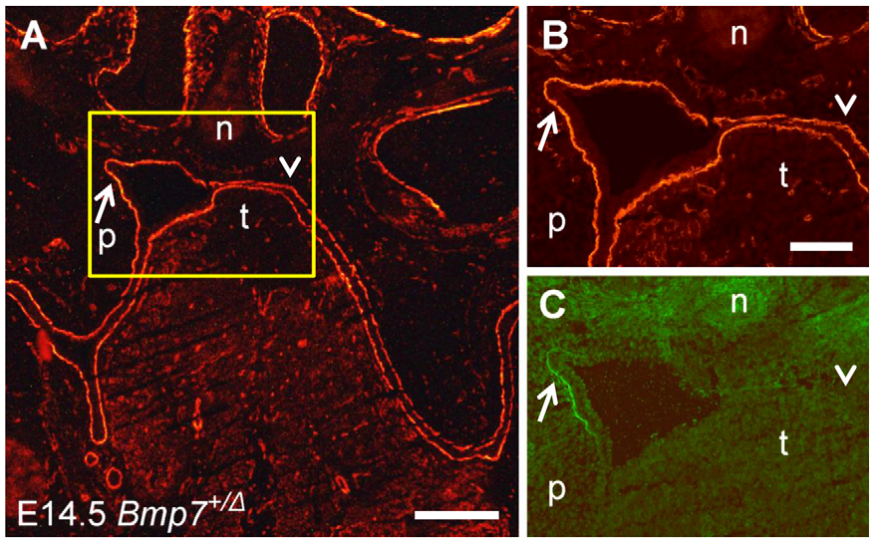
Double labeling for gelatinolytic activity and laminin in the palatal region of a *Bmp7^+/Δ^* mouse embryo. A frontal cryosection of a E14.5 *Bmp7^+/Δ^* mouse head was subjected to DQ-gelatin zymography, followed by immunofluorescence labeling for laminin-111 on the same section. The asymmetric and delayed shelf elevation process provides the possibility for comparing opposing palatal shelves that are at different stages of elevation. (A) Labeling for laminin indicating the region (yellow box) that is shown at higher magnification in (B) (laminin staining) and (C) (in situ zymography), respectively. Gelatinolytic activity colocalizes with the basement membrane of the epithelial fold that is created in the process of elevation of the left palatal shelf (arrows). No sign of gelatinolytic activity is detected in the corresponding region of the opposite shelf where elevation has not yet started (arrowheads). n, nasal cartilage; p, palatal shelf; t, tongue. Bar, 200 μm in A, 100 μm in B and C.

The embryo heads were washed in ice-cold PBS, soaked and embedded in Tissue Tek (O.C.T. compound; Sakura Finetek Europe B.V., Zoeterwoude, Netherlands), and frozen on a metal block cooled to −80°C. All tissue was stored at −80°C before sectioning. Serial frontal sections (10–12 µm thick) of the embryo heads were prepared on a Cryocut E cryomicrotome (Reichert-Jung, Leica Microsystems, Heerbrugg, Switzerland), dried at 37°C for 1–5 min, and stored at −80°C before further use.

**Figure 6:**
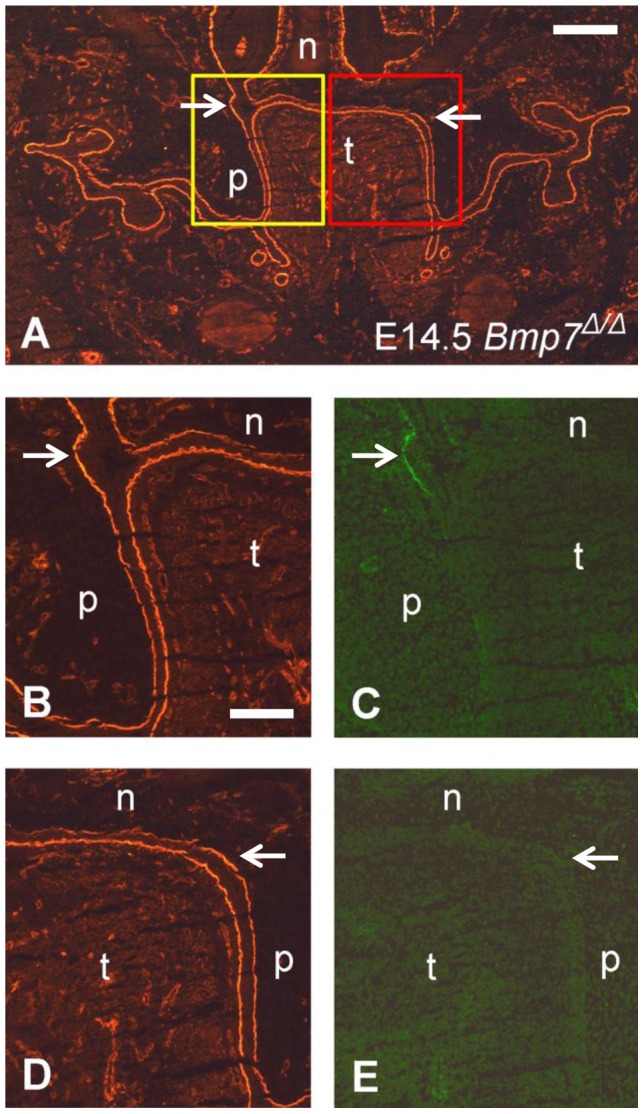
Double labeling for gelatinolytic activity and laminin in the palatal region of a *Bmp7^Δ/Δ^* mouse embryo. A frontal cryosection of a E14.5 *Bmp7^Δ/Δ^* mouse head was subjected to DQ-gelatin zymography, followed by immunofluorescence labeling for laminin-111 on the same section. Note that in the knockout embryo, both palatal shelves are still vertically oriented at this stage. (A) Overview pointing to the regions that are shown in (B–E); laminin staining. The yellow box corresponds to the region that is depicted at higher magnification in (B, C), and the red box to the region in (D, E). (B) Labeling for laminin and (C) in situ zymography, respectively, shows gelatinolytic activity colocalizing with the basement membrane of the epithelial fold that is created at the onset of palatal shelf elevation (arrows); (D) Labeling for laminin and (E) in situ zymography, respectively, did not reveal gelatinolytic activity in the corresponding region of the opposite shelf where no epithelial invagination is visible yet (arrows). n, nasal cartilage; p, palatal shelf; t, tongue. Bar, 250 μm in A, 100 μm in B and E.

### In situ zymography

Fluorescein conjugated, dye-quenched gelatin from pig skin (DQ™-gelatin) was obtained from Molecular Probes (Invitrogen, Basel, Switzerland). A 1 mg/ml stock solution of DQ-gelatin was prepared in gelatinase reaction buffer (150 mM NaCl, 5 mM CaCl_2_, 0.2 mM NaN_3_, 50 mM Tris-HCl, pH 7.6) and stored at 4°C. The working solution for in situ zymography was made by directly diluting DQ-gelatin stock solution in reaction buffer to a final concentration of 20 µg/ml. Unfixed cryosections were thawed, rounded with a wax pen, overlaid with 250 µl DQ-gelatin working solution (for approximately half of the slide), and incubated at 37°C in a dark wet chamber for 3 hours. After three washes with PBS, sections were either processed for immunofluorescence (see below), or mounted directly in 90% glycerol in PBS containing 10 mg/ml propyl 3,4,5-trihydroxybenzoate (Merck, Darmstadt, Germany) as anti-fading agent.

**Figure 7:**
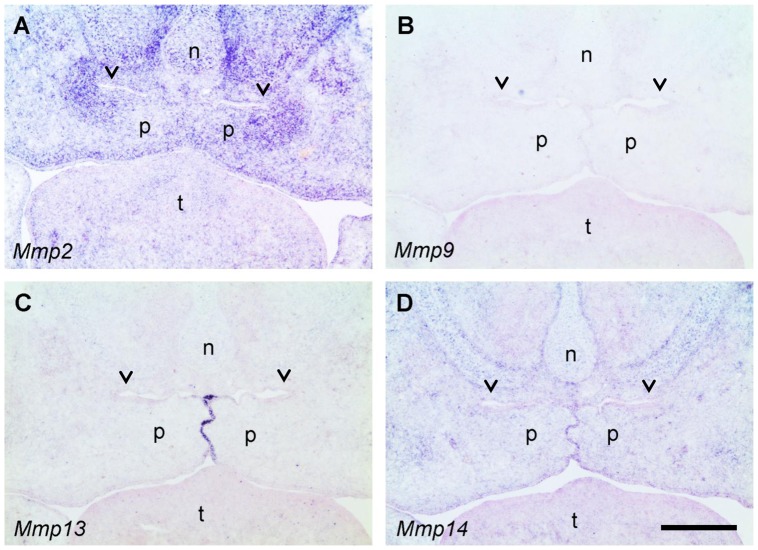
In situ hybridization for MMPs in the mid-palate region of the E14.5 wildtype mouse embryos. Frontal sections were hybridized with antisense RNA probes specific for *Mmp2* (A), *Mmp9* (B), *Mmp13* (C), and *Mmp14* (MT1-MMP) (D), respectively. Blue color indicates specific hybridization; sections have been counterstained with Nuclear Fast Red (pink). Note the strong signal for *Mmp2* mRNA in the mesenchyme around the epithelial fold above the elevated palatal shelves (A), and a weaker signal for *Mmp14* (D). In contrast, no signal for *Mmp9* (B) and *Mmp13* (C) is found in this location (arrowheads); *Mmp13* mRNA is strongly expressed by midline epithelial cells, however. n, nasal cartilage; p, palatal shelf; t, tongue. Bar, 200 μm.

Differences in the extent of gelatinolytic activity during palatal shelf elevation were quantified by measuring the mean pixel intensity (ImageJ Software) in a rectangular area of defined size (20 µm x 50 µm), which included the palatal epithelium and basement membrane of the nasopharyngeal fold, at the middle anteroposterior palatal level. Twelve such regions were measured for each developmental stage (E13.5, E14.5, and E15.5), from a total of 18 mouse embryo heads. Both left and right folds were measured on each section. Background intensity, measured in an adjacent region inside the palatal shelf, was subtracted from the value obtained for each corresponding fold. All measurements were performed twice and the mean value was used for analysis. Non parametric statistics were used for analyzing the results since data were not normally distributed (Shapiro-Wilk test). Statistical analysis was performed through the SPPS Statistics 17.0 software.

**Figure 8:**
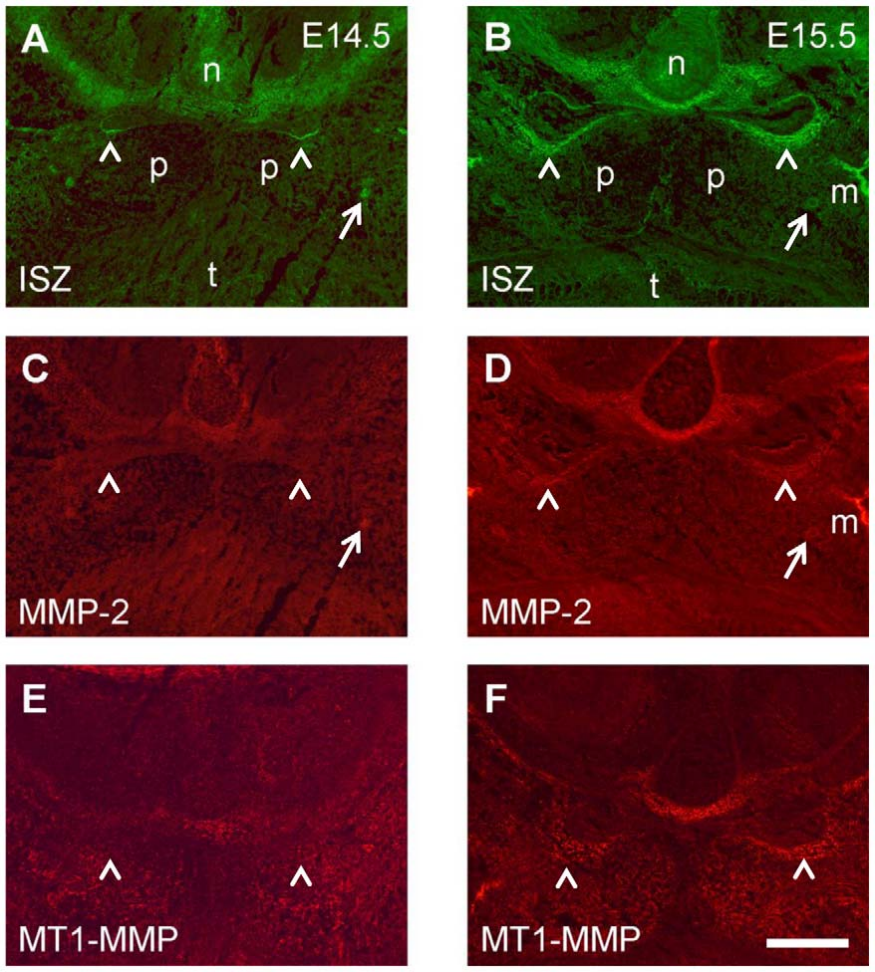
Double labeling for gelatinolytic activity and MMP-2 and MT1-MMP in the palatal region of the mouse embryo. Frontal cryosections of E14.5 (A, C, E) and E15.5 (B, D, F) wild type mouse heads were subjected to DQ-gelatin zymography, followed by immunofluorescence labeling for MMPs on the same section. Serial sections were used; only one zymography image (double labeling for MMP-2) per stage is shown (A, B). Note that despite of differences in distribution, both MMP-2 (C, D) and MT1-MMP (E, F) substantially overlap with gelatinolytic activity, most notably in developing cartilage and bone but also around the expanding nasopharyngeal cavity (arrowheads). For more details, see Results. n, nasal cartilage; p, palatal shelf; t, tongue; m, maxillary bone. Bar, 200 μm.

The specificity of the ISZ protocol used here was thoroughly tested before [33] and confirmed in the present study through various types of control experiments. For negative controls, the DQ-gelatin was either omitted from the gelatinase reaction buffer, or replaced by 20 µg/ml unlabeled pig skin gelatin (Merck); in both cases, no signal was observed. To control for the specificity of the enzyme reaction, either of the following metalloproteinase inhibitors was added to the DQ-gelatin working solution prior to incubation of the slides: 10 mM ethylenediamine tetra-acetic acid (EDTA; Merck; replacing CaCl_2_ in the reaction buffer); 1 mM 1,10-phenanthroline (Phen; Sigma, Buchs, Switzerland); or 50 µM (2R)-[(4-Biphenylylsulfonyl)amino]-N-hydroxy-3-phenylpropionamide (BiPS; MMP-2/MMP-9 inhibitor II; Calbiochem/Merck Chemicals, Nottingham, UK). Addition of either of these reagents to the zymography buffer resulted in partial or complete inhibition of fluorescence generated by DQ-gelatin cleavage. EDTA, a general divalent cation chelator and metalloproteinase antagonist, and phenanthroline, a potent Zn^2+^ complexing agent and MMP inhibitor, almost completely inhibited the gelatinolytic activity. BiPS, a specific MMP-2/MMP-9 inhibitor, significantly attenuated the reaction in situ (Fig. S1).

**Figure 9:**
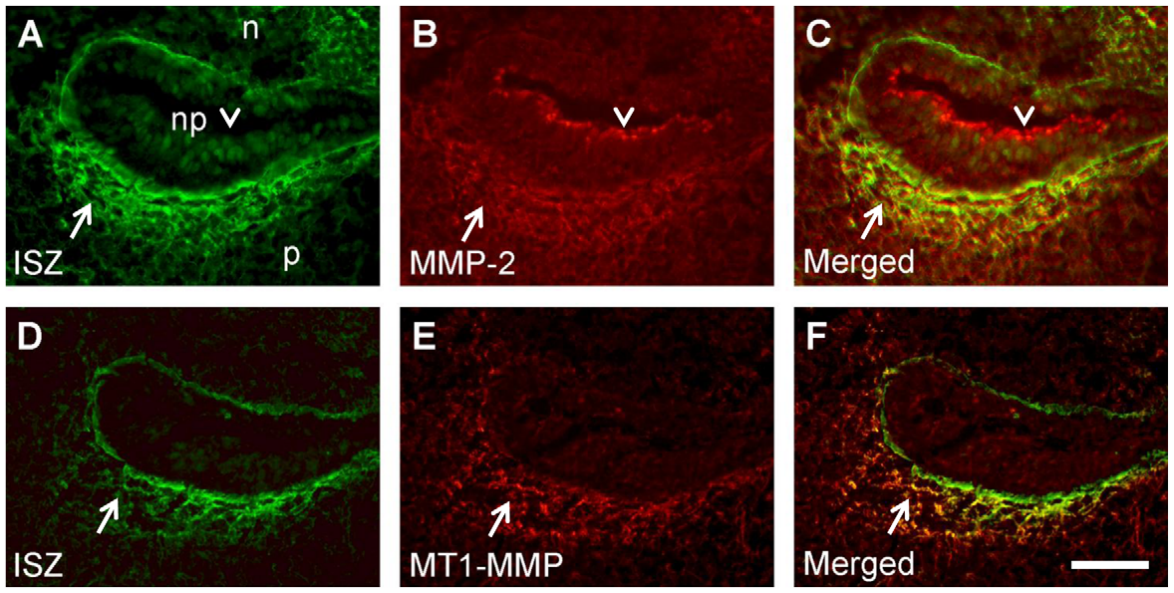
Codistribution of MMP immunostaining with gelatinolytic activity in and around the basement membrane of the palatal fold. Frontal cryosections of E15.5 wild type mouse heads were subjected to DQ-gelatin zymography (A, D), followed by immunofluorescence labeling for MMP-2 (B) or MT1-MMP (E) on the same section. Merged images are shown in C and F. Note colocalization of MMPs with gelatinolytic activity in and around the basement membrane of the epithelial fold (arrows) that is formed above the palatal shelf after its elevation. MMP-2 expression is also evident at the luminal surface of the epithelium where there is no gelatinolysis (arrowheads). For more details, see Results. p, palatal shelf; n, nasal floor; np, nasopharynx. Bar, 50 μm.

### Immunofluorescence staining

To combine ISZ with antibody labeling, we performed ISZ on unfixed cryosections, before continuing with immunolabeling as published previously [33]. Immediately after processing for ISZ, sections were fixed with cold acetone (−20°C) for 1 min, blocked with 3% bovine serum albumin in PBS (BSA/PBS) for 10 min, and then incubated with primary antibody diluted in BSA/PBS for 45 min at room temperature. The following primary antibodies were used: rabbit anti-mouse EHS laminin (1∶200; [35]); goat anti-mouse MMP-2 (1∶20; R&D Systems, Abingdon, UK); goat anti-human MT1-MMP/MMP14 (1∶20; R&D Systems). After three washes in BSA/PBS, sections were covered for 30 min with the respective secondary antibody (rhodamine-conjugated goat anti-rabbit IgG from Cappel/MP Biomedicals, Santa Ana, CA; Cy3-conjugated mouse anti-goat IgG from Jackson ImmunoResearch/Milan Analytica, Rheinfelden, Switzerland) diluted 1∶100 in BSA/PBS. After washing in DEPC-treated H_2_O, slides were mounted in buffered glycerol with anti-fading agent (see above).

**Figure 10:**
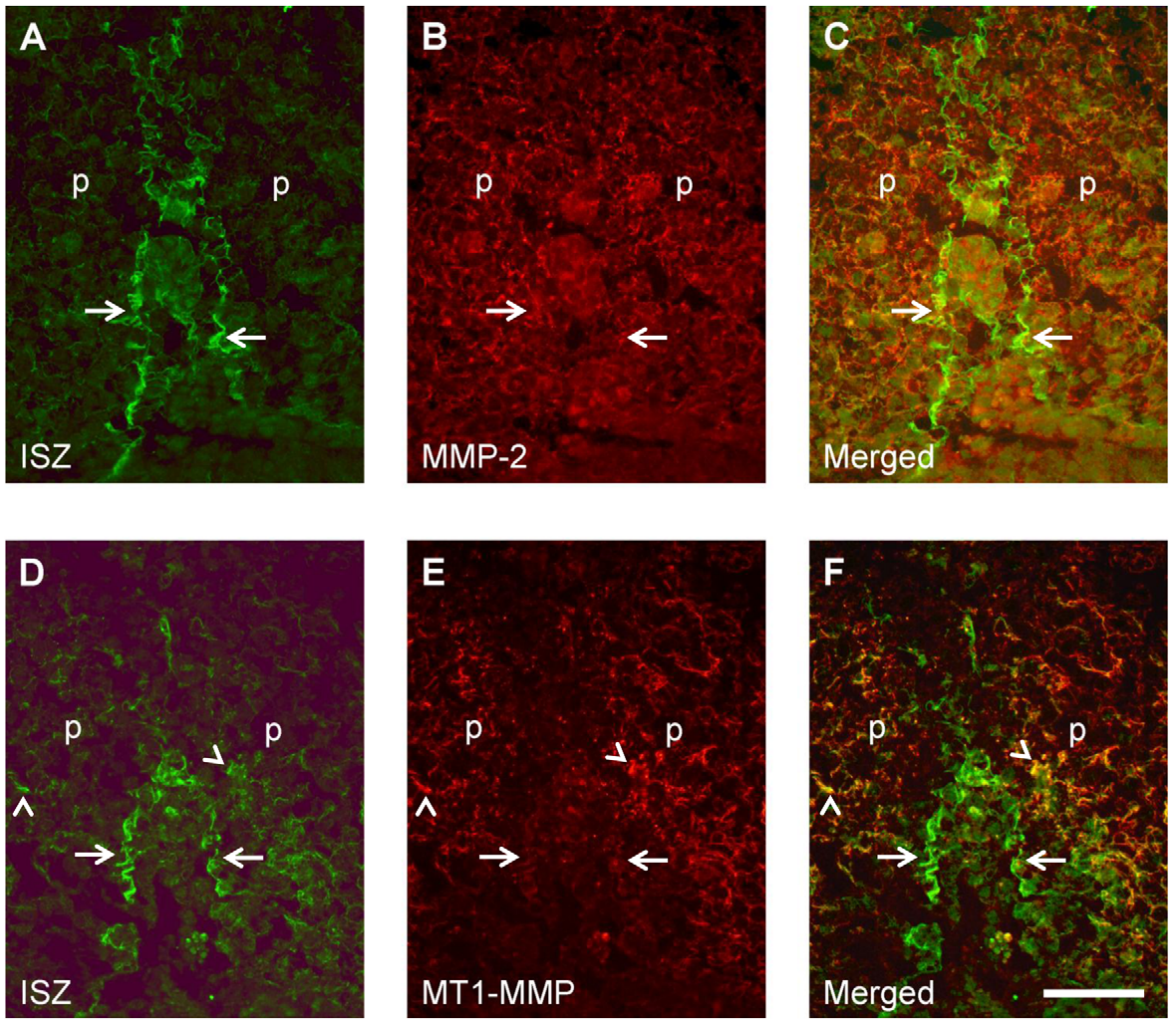
Relationship between MMP immunostaining and gelatinolytic activity at the midline epithelial seam of opposing palatal shelves during their fusion. Frontal cryosections of E15.5 wild type mouse heads were subjected to DQ-gelatin zymography (A, D), followed by immunofluorescence labeling for MMP-2 (B) or MT1-MMP (E) on the same section. Merged images are shown in C and F. At the midline, some colocalization with gelatinolytic activity is evident only for MMP-2 (arrows). MT1-MMP shows weak colocalization with activity at the midline epithelium (arrows) and is more strongly colocalized with gelatinolysis in fibrillar structures adjacent to remnants of the midline epithelium (arrowheads). For more details, see Results. p, palatal shelf. Bar, 50 μm.

### Gene specific RNA probes and in situ hybridization

Total RNA was isolated from E14.5 C57BL/6 wildtype mouse embryos using an RNAeasy Mini Kit (Qiagen, Hombrechtikon, Switzerland) and reverse transcribed to cDNA using Moloney murine leukemia virus reverse transcriptase (Promega, Dübendorf, Switzerland). Primers specific for the murine *Mmp2*, *Mmp9, Mmp*13, and *Mmp14* (MT1-MMP) genes were designed using a program provided by NCBI (http://www.ncbi.nlm.nih.gov/tools/primer-blast/index.cgi?LINK_LOC=BlastHome) and fitted with BamH1 (forward primers) or HindIII (reverse primers) at their 5′ ends, respectively (Table S1). Using these primers (Microsynth, Balgach, Switzerland) and mouse cDNA as a template, specific products were amplified by PCR using Go Taq polymerase (Promega), cut with respective restriction enzymes, and cloned into pBluescript SK+ plasmid (Stratagene/Agilent, Santa Clara, USA). Digoxygenin-labeled anti-sense and sense RNA probes were generated with a labeling kit from Roche Diagnostics [36]. The labeled probes were used for in situ hybridization as published in detail elsewhere [37].

### Microscopy

Slides were viewed with 2x, 4x, 10x, and 40x fluorescence objectives on a Olympus BX-51 microscope equipped with epifluorescence optics, using mirror units U-MWIBA3 for fluorescein (DQ-gelatin) and U-MWIGA3 for rhodamine and Cy3 (secondary antibodies), respectively. Digital images were recorded using a ProgRes CT3 CMOS camera and ProgRes Capture Pro software (Jenoptik, Jena, Germany). For each objective, all slides from one experiment were photographed at exactly the same camera settings, and resulting images were processed identically.

## Results

### Distribution of gelatinolytic activity during palatal morphogenesis in wild type mouse embryos

To present an overview on the relevant anatomical structures, Fig. 1 (A, B) presents frontal cryosections through the entire head of E13.5 and E14.5 mouse embryos, respectively. The sections were labeled with antibody to laminin-111 [35], an integral component of embryonic basement membranes, which also delineates the entire oral epithelium. The staining clearly demonstrates the vertical orientation of the two palatal shelves on either side of the tongue in the E13.5 mouse embryo head, while one day later, at E14.5, the shelves have already elevated above the tongue to form the secondary palate, which separates the oral from the newly generated nasopharyngeal cavity (Fig. 1B). The schemes in Fig. 1C, D indicate different anteroposterior section planes through the palatal region of E13.5 and E14.5 mouse heads, respectively, which are depicted in the following figures.

A double-labeling technique comprising in situ zymography (ISZ) with dye-quenched (DQ-)gelatin, followed by brief acetone fixation and immunofluorescence labeling, was performed to associate gelatinolytic activity in situ with laminin-111 as a marker for embryonic basement membranes (Fig. 2). In the region of the developing secondary palate, no site with prominent gelatinolytic activity was identified prior to palatal shelf elevation at E13.5 at three anteroposterior levels (Fig. 2A–C). In contrast, after palatal shelf elevation at E14.5, gelatinolytic activity was evident in the nasal cartilage and in the two main palatal arteries (Fig. 2D–F). Interestingly, at this embryonic stage signs of gelatinolysis were evident at the midline epithelial seam (MES) formed prior to palatal shelf fusion, and in addition prominent activity was detected as sharp profiles surrounding distinct epithelial structures, such as the folds of the elevated palatal shelves, which contribute to morphogenesis of the nasal cavities in the front and the nasopharynx more posteriorly. In the posterior region, gelatinolytic activity faded out at the dorsal side of the palate. This pattern was also observed at E15.5 stage (Fig. 2G–I), although activity was increased compared to E14.5. Furthermore, prominent activity was evident for maxillary bone, as has been shown before for mandibular bone [33]. The present findings by ISZ of a temporal increase in gelatinolytic activity during embryonic development of the craniofacial region confirm previous results obtained with extracts from mouse embryo heads, which were analyzed for MMP activity (by SDS-gel zymography), protein (by immunoblotting), and mRNA (by RT-PCR), respectively [38].

The extent of gelatinolytic activity at the nasopharyngeal fold was quantified by measuring the integrated pixel intensity in ISZ images of the palatal epithelium and basement membrane over a fixed area. An approximately 5-fold increase was measured from E13.5 to E14.5, and another 3-fold increase from E14.5 to E15.5. Non parametric tests identified significant differences among all groups (Kruskal-Wallis test, p<0.001) and between all pairs of groups compared (Mann-Whitney test, p<0.001), confirming the qualitative observations.

Higher magnification images of the midline epithelial seam (MES) region demonstrated that the appearance of gelatinolytic activity was tightly linked to the process of palatal shelf fusion (Fig. 3). At E13.5, when the palatal shelves were still vertically oriented, no sign of gelatinolysis was detected along the entire oral epithelium, including the prospective fusion sites on the distal-medial aspect of the shelves. Traces of gelatinolysis were evident only within the tongue mesenchyme (Fig. 3A, B). At E14.5, immediately prior to fusion of the palatal shelves, gelatinolytic activity was present at the MES and was located mainly in the pericellular space surrounding a subset of epithelial cells (Fig. 3C, D). At E15.5 during palatal shelf fusion, prominent gelatinolytic activity was associated mainly with remnants of basement membranes of the disorganizing MES (Fig. 3E, F).

ISZ/laminin double labeling also revealed extensive colocalization of gelatinolysis with the basement membranes of the nasopharyngeal epithelial folds that are generated upon palatal shelf elevation (Fig. 4). At stage E15.5 (Fig. 4B, D, F), gelatinolytic activity was also associated with palatal and nasal epithelial cells, as well as with the mesenchyme adjacent to the strongly labeled epithelial basement membranes. Interestingly, activity was strongest at the lateral bend where the oral epithelium folds back on itself, but faded out towards more central regions of the nasopharyngeal cavity. Gelatinolysis was asymmetric and more prominent on the palatal aspect of the folds, in epithelial cells as well as basement membranes and adjacent palatal mesenchyme (Fig. 4A, B).

### In situ zymography during palatal morphogenesis in Bmp7 deficient mice

During normal mouse embryogenesis, elevation of the palatal shelves occurs very rapidly, i.e. within only a few hours on the 14th day of development. To determine whether gelatinolytic activity appeared on the proximal-medial aspect of the palatal shelves already before their elevation, or only afterwards as a consequence of formation of the nasopharyngeal fold, we studied *Bmp7*-deficient embryos. In the absence of Bmp7, palate shelf elevation is delayed and asymmetrical, which eventually leads to cleft palate [39]. Because of asymmetrical palatal shelves, comparisons between different elevation stages and orientations were possible.

In the representative example of a E14.5 *Bmp7* ^wt/Δ^ mouse embryo shown in Fig. 5, gelatinolysis was detected at similar sites as in wild type embryos of the same developmental stage. However, only one of the palatal shelves had just started to elevate (Fig. 5A). Double staining on the same section revealed that gelatinolytic activity codistributed perfectly with laminin-111 immunostaining at the forming epithelial fold of the elevating shelf, although the body of this shelf was still almost vertically oriented. In the same embryo, no sign of gelatinolysis was evident on the opposing side where shelf elevation had not started yet (Fig. 5B, C).

In *Bmp7* ^Δ/Δ^ mouse embryos, both palatal shelves are still vertical at E14.5. The pattern of gelatinolytic activity was comparable to *Bmp7* ^wt/Δ^ embryos of the same stage. However, the even longer delay in elevation revealed that gelatinolysis started already in vertically oriented shelves at the onset of the process. In the example shown in Fig. 6, early formation of a nasopharyngeal fold is visible as a notch in the epithelial basement membrane of the left palatal shelf, and this site was clearly positive for gelatinolytic activity (Fig. 6B, C). The opposing shelf, where the elevation process had not started yet, did not present any activity at the corresponding location (Fig. 6D, E).

These findings indicate that gelatinolytic activity first appears in the basement membrane of the vertical palatal shelf at exactly the place and time when the nasopharyngeal fold starts forming, and immediately before the actual process of shelf elevation is initiated.

### MMP mRNA expression in the palate region

Besides the major gelatinases MMP-2 and MMP-9, collagenase-3 (MMP-13) and the membrane-bound MT1-MMP (MMP-14) also have considerable gelatinolytic activity [20], [40] and are expressed in developing craniofacial structures [12]. We therefore asked which of these four genes are expressed around the nasopharyngeal folds that form upon palate elevation at E14.5. In situ hybridization showed a strong enrichment for *Mmp2* mRNA in the mesenchyme surrounding the palatal folds (Fig. 7A). A weak but specific signal was also observed there for *Mmp14* (MT1-MMP; Fig. 7D). In contrast, mRNA for *Mmp9* could not be detected in the entire palate at this stage (Fig. 7B), although the probe strongly labeled scattered cells (presumably osteoclast precursors) within developing mandibles on the same section (not shown). Like *Mmp14* (Fig. 7D), *Mmp13* was specifically expressed by midline epithelial cells (Fig. 7C) as has been demonstrated before [12], but no *Mmp13* signal was detected in or near the epithelium of the palatal folds.

### Colocalization of gelatinolytic activity with immunolabeling for MMP-2 and MT1-MMP in the developing palate

MMPs are secreted as latent proenzymes that require enzymatic processing for activation [19]; thus the distributions of latent and active gelatinases might differ. The membrane-bound MT1-MMP is known to activate other MMPs, particularly MMP-2, and itself possesses gelatinolytic activity [20]. Since these were the two MMPs found to be prominently expressed in the palatal region, we employed ISZ followed by immunohistochemistry on sections of E13.5, E14.5, and E15.5 wildtype mouse heads, in order to investigate the relationship between gelatinolytic activity and enzyme distribution (Fig. 8).

MMP-2 and MT1-MMP showed weak but almost ubiquitous protein expression in the palatal region of the mouse wildtype embryo at E13.5 (not shown). At E14.5, MMP-2 protein was widely distributed in the nasal and palatal region (Fig. 8C). Highest expression was noted in the perichondrium of the nasal cartilage, around the main palatal arteries, and weakly around the newly formed nasopharyngeal epithelial folds above the palate, where gelatinolytic activity was also observed (Fig. 8A, C). Whereas MMP-2 immunostaining overlapped with gelatinolytic activity at many sites, latent MMP-2 appeared to be enriched in perichondrium of nasal cartilage, where enzyme activity appeared lower than in the cartilage proper. At E15.5, the pattern of MMP-2 distribution remained similar, although the amount of protein was increased, as was gelatinolytic activity (Fig. 8B, D). MMP-2 colocalized with strong gelatinolysis at the basal side of the nasal septum, in the newly formed maxillary bone (as has been shown before for mandibular bone; [33]), and in the palatal mesenchyme adjacent to the nasopharyngeal fold. MT1-MMP immunoreactivity was also prominent at these sites (Fig. 8E, F). However, this enzyme had a more restricted expression than MMP-2 since it was almost absent from the cartilage proper but enriched in perichondrium (Fig. 8F), in accordance with its mRNA distribution (c.f. Fig. 7D). Weak but clear staining was observed throughout the palatal shelves.

Higher magnification double labeling images revealed extensive colocalization of MMP-2 and MT1-MMP with gelatinolytic activity around the nasopharyngeal fold above the palate at E15.5 (Fig. 9). MMP-2 protein overlapped with activity both in epithelial basement membrane and palatal mesenchyme; in addition significant amounts of latent MMP-2 were evident at the luminal surface of the epithelium where no activity was detected (Fig. 9A–C). MT1-MMP colocalized with activity primarily in palatal mesenchyme adjacent to the epithelial fold (Fig. 9D–F). Thus, the asymmetric distribution of gelatinolytic activity around the nasopharyngeal fold (c.f. Fig. 4) was matched by MMP-2 and MT1-MMP expression patterns.

In contrast, gelatinolytic activity associated with the fusing midline epithelial seam of E15.5 palatal shelves (c.f. Fig. 3) only loosely correlated with MMP-2 and MT1-MMP immunostaining (Fig. 10). The almost ubiquitous MMP-2 codistributed in only a few places with gelatinolytic activity at the disintegrating midline basement membranes (Fig. 10A–C). MT1-MMP appeared to be associated with gelatinolysis in a subset of mesenchymal fibrils adjacent to remnants of the midline epithelium (Fig. 10D–F).

In summary, whereas gelatinolytic activity extensively codistributed with immunoreactivity for MMP-2 and MT1-MMP around the nasopharyngeal fold that forms above the palate, no such clear correlation was found at the fusing midline epithelial seam.

### Gelatinolytic activity associated with basement membranes of other folding epithelia in the developing orofacial region

Apart from the palatal region, gelatinolytic activity was also detected in epithelial basement membranes in other regions of the developing mouse head by in situ zymography. In the middle of the snout of the E15.5 mouse, increased gelatinolytic activity was detected not only in the nasopharyngeal folds, but also at the basolateral aspect of the newly formed nasal cavities, and in the developing nasal turbinates at the lateral borders of the nasal cavities (Fig. S2A). Furthermore, double staining for enzymatic reaction and laminin-111 showed prominent gelatinolytic activity in a section of epithelial basement membrane of the lower lips, anteriorly besides the tongue (Fig. S2B, C), as well as more posteriorly around the lateral margins of the oral cavity that are defined by the folding oral epithelium (Fig. S2D, E). In all three cases described above, a similar pattern of gelatinolysis was detected also at E14.5, although the intensity of activity was lower at the earlier stage. At E13.5, no activity was present yet at any of these sites (not shown).

## Discussion

Morphogenesis of the secondary palate in mammals involves a sequence of essential steps, among them the elevation of the two palatal shelves from a horizontal to a vertical position above the tongue, followed by their fusion at the midline [7], [8]. Whereas palatal shelf fusion has been extensively studied, much less is known about the mechanism of shelf elevation, which happens in an amazingly short time frame [7]. In the normal mouse embryo, the process occurs within less than a day and requires that the tongue moves out of the way of the rising shelves. In embryos defective for the *gad67* gene, palatal shelves elevate within only 30 minutes after the tongue has been removed experimentally [41]. It has long been speculated how and where the forces for this rapid reorientation of palatal shelves are generated. A prominent hypothesis proposed a rotation of the shelves in a “barndoor” fashion, thought to be driven by hyaluronic acid-generated osmotic pressure [8], [26], [38]. However, a causative relationship between hyaluronan accumulation and shelf elevation has never been established, and such a mechanism might be difficult to reconcile with the fast kinetics of the process. In fact, the simple rotation model of palatal shelf elevation has been challenged in recent years. Chou et al. [29] used carbon markings to produce fate maps of fetal mouse palates during elevation in culture. From their data, the authors concluded that palatal shelves primarily bulge out mesially in their anterior and posterior regions during elevation, whereas some rotation is involved in the mid-palate region. A more recent histomorphometric study confirmed these findings by showing that the horizontal outgrowth of vertically oriented palatal shelves starts from the medial side of the mid-posterior region, and palatal shelf reorientation occurs in a dynamic, spatiotemporally coordinated manner along the AP axis [42]. In another recent study, Jin et al. [28] demonstrated that the prospective medial edge is not located at the very distal rim of vertical palatal shelves, but instead more medially, on their interior aspect facing the tongue. Using a mouse mutant (*Zfhx1a^−/−^*) in which palatal shelf elevation is retarded by one day, they found that genes typical for the prospective medial edge (*Mmp13* for the epithelium and *Goosecoid* for the mesenchyme) were expressed at the distal-medial (lingual) aspects of the still vertically positioned shelves, rather than at their distal tips [28].

From these newer findings, it seems evident that palate elevation is caused by rapid and extensive tissue reorientation and remodeling within and perhaps around the shelves. Tissue reorganization by coordinated cell movements can occur very rapidly and is widespread in animal morphogenesis [30]. Mesial bulging of palatal shelves (towards the tongue) might be based on coordinated, actomyosin-based cell contraction [30] in the dorso-ventral direction, but such a mechanism has yet to be demonstrated. In addition to bulging, it appears that the dorso-medial corner of the shelves retracts laterally during elevation, in order to generate the necessary space for the developing nasopharyngeal cavity. In E13.5-14.0 wildtype embryos, a first sign of tissue retraction is observed as a slight epithelial invagination on the upper lingual aspect of palatal shelves, but it is more obvious at a slightly later stage in *Bmp7* mutant embryos, in which shelf elevation is delayed [39]. This “notch” in the oral epithelium eventually develops into the fold that forms the lateral margin of the emerging nasopharyngeal cavity. Tissue reorganization during morphogenesis often requires remodeling of the surrounding extracellular matrix, and a role for gelatinases and other MMPs has been established for several morphogenetic processes during mouse embryogenesis [43]. The present study demonstrates for the first time the appearance of gelatinolytic activity in elevating palatal shelves, specifically in the basement membrane and adjacent mesenchyme at the exact site of epithelial invagination. Since gelatinolysis at this site can be observed even shortly before elevation, it does not appear to be a consequence of shelf reorientation, but instead might be causally involved in the process.

A recent study showed that prior to palatal shelf elevation, *Fgfr2* is highly expressed at the nasal aspect of the palatal shelves where the epithelial fold will be formed, and where also increased gelatinolytic activity was detected by our study. *Fgfr2* mutant mice present with a cleft phenotype due to delayed shelf elevation, which is attributed to the decreased cellular proliferation and decreased ECM accumulation near the time of elevation [44]. This strengthens our hypothesis that increased ECM remodeling at this site is important for palatal shelf elevation. The importance of directional cell migration and proliferation in palatal mesenchyme during normal elevation, which also implies increased ECM remodeling, has also been elucidated by other recent studies using different knockout models (for review see [45]).

After the nasopharyngeal cavity has formed above the secondary palate at E14.5, gelatinolytic activity remains associated precisely with the lateral margin of the epithelial fold, and even increases at E15.5. We propose that MMP activity is required for extracellular matrix turnover and for tissue retraction occurring in the dorsal-medial region of the elevating palatal shelves [28]. This is indicated by the fact that gelatinolytic activity and MMP expression appear more pronounced on the palatal (lower) than on the nasal (upper) side of the epithelial fold (c.f. Fig. 3). The observed increase in gelatinolysis around the epithelial folds at E15.5 might be required to keep its lateral margins in place despite of a rapidly growing tissue environment. We thus hypothesize that MMP activity also prevents the nasopharyngeal cavity from being closed during growth of the maxilla and the secondary palate.

Interestingly, we found additional examples of forming epithelial invaginations in the mouse embryo head that were associated with strong gelatinolytic activity. In the oral cavity, activity was detected around the deep fold that separates the base of the tongue from the mandible [33], and also at its most lateral edge that separates the upper from the lower jaw. In the nasal cavity, prominent gelatinolysis was present around the epithelial crypts between the developing nasal turbinates. Including the nasopharyngeal cavity above the palate, these are all cases where epithelia bend back on themselves to form crypt-like structures, and where the extreme ends of cavities need to be maintained and expanded during later stages of morphogenesis. Thus, one might speculate that localized expression of MMP activity is part of a more general mechanism to generate and stabilize epithelial crypts in a rapidly growing organism. This hypothesis is further supported by the fact that apart from basement membranes, gelatinolytic activity appears to be increased also above the epithelium itself at these sites. According to recent evidence, MMP function has been associated with epithelial cell motility or even apoptosis [46], [47], [48], but this has to be further investigated.

The importance of basement membrane remodeling and the role of MMP-2 and activating enzymes, like MT1-MMP, in this process has been shown in various developmental and cancer studies [49]. The contribution of MMP-2 in this process is attributed to the degradation of type IV collagen, a major component of the basement membrane [50]. We asked which MMPs might be responsible for the gelatinolytic activity around the epithelial folds of the forming nasopharyngeal cavity above the secondary palate. In situ hybridization experiments revealed that of the two major gelatinases, MMP-2 but not MMP-9 was strongly and specifically expressed at these sites. In addition, MT1-MMP, a cell membrane-bound enzyme that is required for the activation especially of pro-MMP-2 [20], showed a weaker but similar mRNA distribution. Immunolabeling confirmed that MMP-2 and MT1-MMP protein colocalized extensively with the pattern of gelatinolysis around the epithelial folds of the palate. We previously showed codistribution of gelatinolytic activity with these MMPs around the crypt that separates the developing tongue from the mandibles [33]. In contrast to these epithelial folds, however, we detected hardly any codistribution of gelatinolytic activity with MMP-2 and MT1-MMP proteins at the midline epithelial seam during palate fusion, although both enzymes were detected in the adjacent palatal mesenchyme where they partially colocalized with activity. Since antibodies also recognize inactive proenzymes, it is to be expected that MMP proteins and gelatinolytic activity do not overlap perfectly. Nevertheless, we assume that other MMPs are responsible for the observed gelatinolysis at the MES. The most likely candidate is MMP-13 (collagenase-3), which also cleaves denatured fibrillar collagen (gelatin) [40] and is expressed by midline epithelial cells (Fig. 6; [12]). The expression of MMP 13 in the MES has been implicated before in palatal shelf fusion [12]. Although *Mmp13* knockout mouse do not present with a cleft phenotype [51], MMP-13 is the main candidate responsible for the detected activity in MES. This is strengthened by evidence that MMP-13 forms complexes with MMP-2 and MT1-MMP, resulting in synergism between these proteases [52]. Thus, the presence of all three MMPs in and adjacent to the MES, as demonstrated by in situ hybridization and immunohistochemistry here, might be important for palatal shelf fusion.

In summary, our present study implies a function for MMP-dependent gelatinolysis not only in palatal shelf fusion as has been suggested before, but for the first time also in the process of palatal shelf elevation/reorientation. Specifically, gelatinolytic activity was detected in the epithelial fold and retracting mesenchyme at the upper medial corner of palatal shelves, exactly at the time of their reorientation. Colocalization studies were consistent with the notion that MMP-2 is primarily responsible for gelatinolytic activity at this site, and that MT1-MMP might also be involved either directly, or indirectly through activation of MMP-2. However, additional experiments are required to establish a causal relationship.

## Supporting Information

Figure S1 **Effect of MMP inhibition on development of fluorescent signal during in situ zymography with DQ-gelatin.** Unfixed frontal cryosections of E14.5 wild type mouse heads were incubated with DQ-gelatin (see Materials and Methods), with or without adding inhibitors to the reaction buffer. (A) Control without inhibitor. (B) Inclusion of a specific MMP-2/MMP-9 inhibitor (BiPS; 50 µM; diluted in 0.2% DMSO) partially attenuated the reaction. (C) General MMP inhibitors 1,10-phenanthroline (1 mM) and (D) EDTA (10 mM) strongly suppressed the gelatinolytic activity. (E) Incubation of slides with only ISZ buffer (without DQ-gelatin) or (F) replacement of DQ-gelatin by 20 μg/ml of unlabeled pig skin gelatin did not produce any fluorescent signal. n, nasal cartilage; p, palatal shelf; t, tongue. Bar, 200 μm.
(TIF)

Figure S2 **Gelatinolytic activity associated with distinct epithelial structures at other sites of the E15.5 mouse embryo head.** Frontal cryosections of E15.5 wild type mouse heads were subjected to DQ-gelatin zymography, followed by immunofluorescence labeling for laminin on the same section. (A) In situ zymography of a section in the middle of the snout showing increased gelatinolytic activity in the epithelial folds formed at the lower lateral part of the nasal cavities created after palatal shelf elevation, and in the developing nasal turbinates (arrows). (B) In situ zymography and (C) immunofluorescence labeling for laminin, respectively, of the lateral side of the mouth opening. Note the presence of gelatinolytic activity at the epithelial basement membrane of the lower lip next to the tongue (arrows). (D) In situ zymography and (E) immunofluorescence labeling for laminin, respectively, of the lateral limits of the oral cavity. Prominent gelatinolytic activity is evident at the epithelial basement membrane of the fold that is created at the lateral end of the oral epithelium separating upper and lower jaw (arrows). n, nasal cartilage; nc, nasal cavity; p, palatal shelf; nt, nasal turbinates; up, upper lip; lo, lower lip; utb, upper tooth bud; ltb, lower tooth bud. Bar, 250 μm in A, 100 μm in B–E.
(TIF)

Table S1 (DOC)
