# Supplementary figures and images for: Site-Specific Expression of Gelatinolytic Activity during Morphogenesis of the Secondary Palate in the Mouse Embryo
Source: PLoS One. 2012 Oct 16;7(10):e47762. doi: 10.1371/journal.pone.0047762 (PMC3472992; doi:10.1371/journal.pone.0047762)

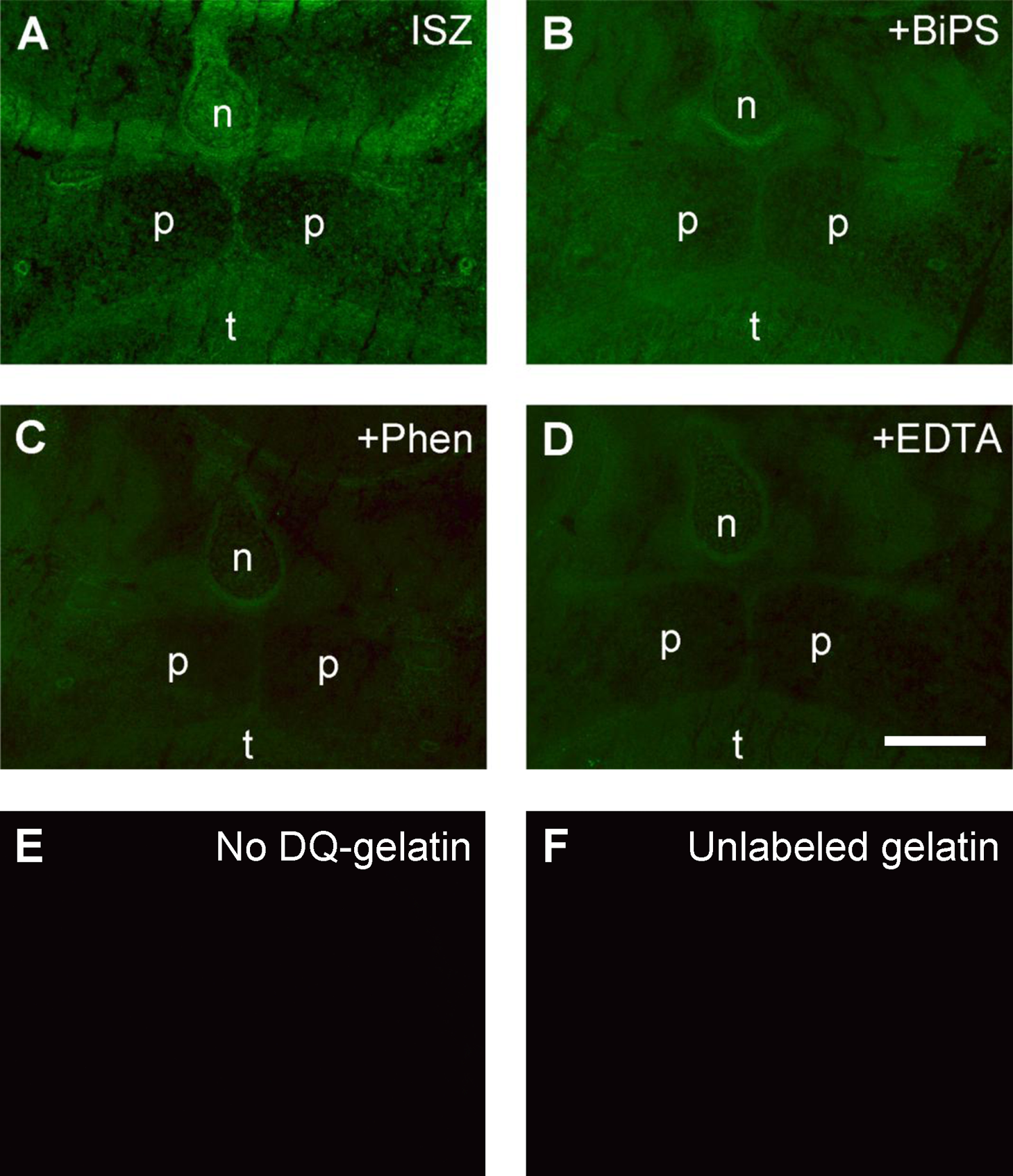

Supplement: Figure S1 — Effect of MMP inhibition on development of fluorescent signal during in situ zymography with DQ-gelatin. Unfixed frontal cryosections of E14.5 wild type mouse heads were incubated with DQ-gelatin (see Materials and Methods), with or without adding inhibitors to the reaction buffer. (A) Control without inhibitor. (B) Inclusion of a specific MMP-2/MMP-9 inhibitor (BiPS; 50 µM; diluted in 0.2% DMSO) partially attenuated the reaction. (C) General MMP inhibitors 1,10-phenanthroline (1 mM) and (D) EDTA (10 mM) strongly suppressed the gelatinolytic activity. (E) Incubation of slides with only ISZ buffer (without DQ-gelatin) or (F) replacement of DQ-gelatin by 20 μg/ml of unlabeled pig skin gelatin did not produce any fluorescent signal. n, nasal cartilage; p, palatal shelf; t, tongue. Bar, 200 μm. (TIF) [file pone.0047762.s001.tif]

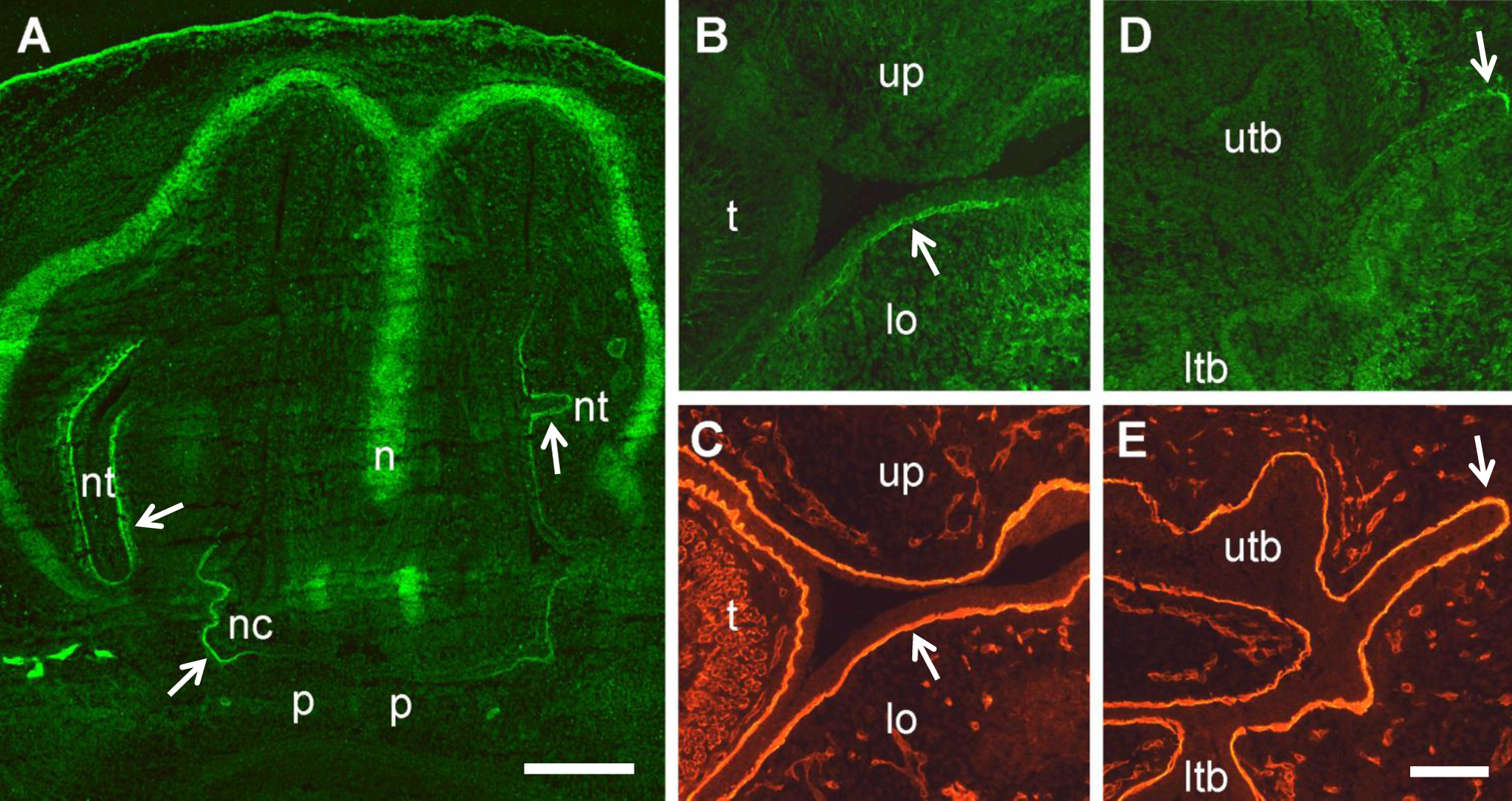

Supplement: Figure S2 — Gelatinolytic activity associated with distinct epithelial structures at other sites of the E15.5 mouse embryo head. Frontal cryosections of E15.5 wild type mouse heads were subjected to DQ-gelatin zymography, followed by immunofluorescence labeling for laminin on the same section. (A) In situ zymography of a section in the middle of the snout showing increased gelatinolytic activity in the epithelial folds formed at the lower lateral part of the nasal cavities created after palatal shelf elevation, and in the developing nasal turbinates (arrows). (B) In situ zymography and (C) immunofluorescence labeling for laminin, respectively, of the lateral side of the mouth opening. Note the presence of gelatinolytic activity at the epithelial basement membrane of the lower lip next to the tongue (arrows). (D) In situ zymography and (E) immunofluorescence labeling for laminin, respectively, of the lateral limits of the oral cavity. Prominent gelatinolytic activity is evident at the epithelial basement membrane of the fold that is created at the lateral end of the oral epithelium separating upper and lower jaw (arrows). n, nasal cartilage; nc, nasal cavity; p, palatal shelf; nt, nasal turbinates; up, upper lip; lo, lower lip; utb, upper tooth bud; ltb, lower tooth bud. Bar, 250 μm in A, 100 μm in B–E. (TIF) [file pone.0047762.s002.tif]
