# Supplementary material for: Site-Specific Expression of Gelatinolytic Activity during Morphogenesis of the Secondary Palate in the Mouse Embryo
Source: PLoS One. 2012 Oct 16;7(10):e47762. doi: 10.1371/journal.pone.0047762 (PMC3472992; doi:10.1371/journal.pone.0047762)
Supplement: Table S1 — (DOC) [file pone.0047762.s003.doc]

**Table S1: Primers used for generation of gene-specific RNA probes for in situ hybridization**

| **Gene** | **Direction** | **Primer sequence** |
| --- | --- | --- |
| Mouse *Mmp2*: | Forward: | CCGGATCCGGGAGAAGGACAAGTGGTCCGC |
|  | Reverse: | CCAAGCTTCACCTTGCCATCGTTGCGGC |
| Mouse *Mmp9*: | Forward: | CCAAGCTTCCTTACCAGCGCCAGCCGAC |
|  | Reverse: | CCGGTACCAGAAGGCCGTCCTTGCCG |
| Mouse *Mmp13*: | Forward: | CCGGATCCATTCAGCTATCCTGGCCACCTTC |
|  | Reverse: | CCAAGCTTCATCCACATGGTTGGGAAGTTCTG |
| Mouse *Mmp14*: | Forward: | CCGGATCCATGATGGCCATGAGGCGCC |
|  | Reverse: | CCAAGCTTGGGCCCATAGGCGGGGTT |
